# Cross-border supply chain coordination of low-carbon agricultural products under the risk of supply uncertainty

**DOI:** 10.1371/journal.pone.0309763

**Published:** 2024-10-22

**Authors:** Zheng Liu, Na Huang, Bin Hu, Wenzhuo Sun, Lihua Shi, Yuanjun Zhao, Chunjia Han

**Affiliations:** 1 School of Management, Shanghai University of Engineering Science, Shanghai, China; 2 School of Accounting, Nanjing Audit University, Nanjing China; 3 Birkbeck Business School, Birkbeck, University of London, London, United Kingdom; Ankara Yildirim Beyazit University / Worcester Polytechnic Institute, TÜRKIYE

## Abstract

The purpose of this paper is to discuss how cross-border e-commerce enterprises can promote the sustainable development of the supply chain by optimizing the risk of supply disruption and product quality control mechanism of the cross-border supply chain of low-carbon agricultural products in the face of the problem of uneven quality and inventory shortage that prevails in the supply chain of low-carbon agricultural products under the framework of low-carbon economy. Methods: A two-level supply chain model consisting of a risk-averse cross-border e-commerce enterprise and two risk-neutral overseas suppliers is constructed to compare the optimal strategies and their coordination effects under the centralized and decentralized decision-making modes, and to deeply analyze the supply chain’s operation mechanism. Further, the quality cost factor is introduced and an option contract model is designed to quantitatively analyze the impact of different decision-making scenarios and parameter changes on the overall supply chain performance. The results of the study show that under the coordination of option contract, the profit of cross-border e-commerce companies tends to decrease as the risk of supply disruption increases, while the profit of suppliers rises accordingly. Meanwhile, improving the quality of agricultural products can significantly improve the overall profitability of the supply chain. The cost-sharing mechanism is positively related to the profit of cross-border e-commerce companies, but negatively affects the profit of suppliers. In addition, the adjustment of the option price is directly associated with the increase of the specific supplier’s profit and the decrease of the cross-border e-commerce enterprise’s profit. Conclusion: By constructing and analyzing the option contract model considering the risk of supply disruption, this study effectively reveals the key influencing factors and their interactions in the cross-border supply chain of low-carbon agricultural products, and provides cross-border e-commerce enterprises with specific strategies to achieve coordination of the supply chain contract, improve product quality, and mitigate the risk of supply disruption, and then promote the sustainable development of the supply chain of low-carbon agricultural products.

## Introduction

At a time when the trend of global warming is becoming more and more serious, reducing greenhouse gas (GHG) emissions and combating climate change have become global challenges. Agriculture, as one of the main sources of greenhouse gas emissions, has a significant impact on the environment, directly or indirectly, through its production activities. According to the United Nations Intergovernmental Panel on Climate Change (IPCC), GHG emissions from agricultural land account for more than 14% of total global anthropogenic emissions, making it the third largest source of emissions after the energy and building sectors [1]. Agricultural products, as the main products of agriculture, are accompanied by energy consumption and GHG emissions throughout the supply chain from cultivation to consumption, including cultivation, processing, packaging, transportation and distribution [2]. The rising carbon emissions associated with large-scale production and distribution of agricultural products, increased consumer awareness of the concept of green consumption, the sharp increase in global demand for low-carbon agricultural products, and inconsistencies in the quality of agricultural products and inventory fluctuations that increase the risk of disruptions in the supply chain pose serious challenges to the agricultural supply chain. Therefore, in order to ensure the low-carbon sustainable development of the agricultural supply chain, enterprises must deeply integrate low-carbon technologies and concepts into their operations and pay close attention to the risks of product quality fluctuations and supply chain disruptions that may be encountered in the whole chain of low-carbon agricultural products from production to sales. These risks are not only related to the stability of the supply chain, but also directly affect the maximization of environmental and economic benefits [3]. Therefore, under the premise of fully considering the risk of product quality and supply chain disruption, it is particularly important to conduct an in-depth study on the revenue coordination of the cross-border e-commerce import supply chain of low-carbon agricultural products. This not only helps to promote the innovation and upgrading of low-carbon supply chain management of agricultural products, but also significantly improves the added value of agricultural products, which in turn injects new vitality into the vigorous development of low-carbon economy.

In the context of a complex global environment, supply chain stability and efficiency are critical to business success. However, supply chain disruptions are frequent and are usually caused by unpredictable and unexpected events, leading to disruptions in the flow of materials and interruptions in business operations. In order to solve this kind of problem and effectively reduce the risk of supply disruption so as to improve the resilience of the supply chain, related researchers have carried out research from the supply chain main body, product quality, risk factors of these levels. From the perspective of the supply chain subject, in the traditional supply chain, in the face of the risk of supply disruption, the supplier can combine the government subsidy mechanism to develop operational strategies to build the optimal operation and subsidy policy model, to achieve the system optimization [4]. For retailers, supplier reliability is a key factor in making sourcing and pricing decisions to address the risk of supply disruptions. When a retailer enjoys purchasing cost advantages and market advantages it prefers reliable supply, while these advantages are occupied by competitors, it prefers suppliers with higher risk of cheap supply disruption [5]. From the perspective of supply chain product quality, the quality of agricultural products in different stages of the supply chain varies depending on the characteristics of the product and the environment in which it is located, for example, the time of the transportation stage and the temperature at which the transportation is maintained are considered to be important factors affecting its quality [6]. The quality loss of agricultural products in the supply chain has been dynamically estimated in existing studies mainly by setting a quality function [7], so as to restore the real agricultural supply chain situation as much as possible. In addition regular updating of production, product transportation and storage decisions [8], differentiated design of product lines for green and non-green products [9], quality disclosure strategies [10], consumer quality perception [11], quality improvement incentives [12], pricing and refund policies [13], and information sharing [14] have been found to have a greater impact on achieving optimal coordination in the supply chain. From the perspective of supply disruption risk, there are four main types of supply risk, namely demand, process, supply, and supply and environmental disruption risk [15]. Adopting strategies such as increased investment in research and development (R&D), strengthening organizational analytical capabilities, and pre-sales can effectively mitigate the impact of supply disruptions [16,17].

Quality and risk management in agricultural supply chains is more complex and has more uncertainties than traditional supply chains [18], such as seasonality, supply peaks, longer supply lead times and perishability [19]. These factors make agricultural supply chains more sensitive to the risk of supply disruptions, as delays or failures at any point in the chain can lead to a serious impact on the efficiency and reliability of the entire supply chain. For example, a reduction in crop production or a delay in harvesting due to weather changes [20] can quickly trigger supply chain disruptions, affecting market supply and price stability. The disruption of their supply not only causes economic losses, but also increases agricultural greenhouse gas emissions due to the high energy consumption of their cold chain transportation [21,22]. Therefore, in the context of climate change, the main body of the agricultural product supply chain should simultaneously consider the factors of decarbication and risk interruption of the product supply chain when dealing with the risk of supply interruption. Existing studies have shown that increasing the use of renewable energy can effectively promote low-carbon development [23], and establishing a supply chain energy model can alleviate energy consumption [24]. Government subsidies and tax strategies [25] are effective measures to promote the decarbonization of agricultural supply chains.

In summary, the above existing studies have been more in-depth on supply chain disruptions and agricultural supply chain management strategies and supply chain decarbonization, but they mostly focus on pricing and sourcing strategies for the risk of supply disruptions of agricultural products in fresh supply chains or traditional supply chains, decarbonization of agricultural supply chains, and the interactions between price, profit and incentive strategies. This study focuses on how cross-border supply chains face a more complex external environment, and in particular how it is critical for low-carbon agricultural products to simultaneously control disruption risks and quality levels. Therefore, the contribution of this paper is to focus on the cross-border low-carbon agricultural supply chain, consider the interruption risk and quality level, construct the option-quality cost-sharing contract secondary supply chain model, and discuss the optimal decisions of the cross-border supply chain members under different decision scenarios. The use of this paper’s model and research results for cross-border low-carbon agricultural products supply chain to effectively realize the contractual coordination of cross-border e-commerce supply chain, improve the quality level of low-carbon agricultural products, alleviate the problem of supply disruption to provide references and bases, and then promote the sustainable development of low-carbon agricultural products supply chain.

## Methodology

### Model hypothesis and parameter description

This study focuses on the coordination of gains in cross-border supply chains of low-carbon agricultural products, especially the interaction between cross-border e-commerce firms and overseas suppliers in the context of globalized trade. This study constructs a model that simulates a secondary supply chain consisting of a risk-averse cross-border e-commerce firm and two risk-neutral overseas suppliers. In this model, market demand is mainly influenced by the level of quality of low-carbon agricultural products provided by suppliers. The complete sharing of information between cross-border e-commerce companies and overseas suppliers ensures the transparency of the supply chain. Overseas suppliers are in a dominant position due to their market position and technological advantages. All supply chain members, both firms and suppliers, share the common goal of profit maximization. However, cross-border supply chain operations are affected by a variety of factors, including national trade policies, transportation distances and customs clearance processes. These factors result in order lead times for cross-border low-carbon agricultural products often being longer than the sales cycle, increasing supply chain uncertainty. Cross-border e-commerce firms may face the risk of stock-outs when market demand exceeds order quantities. At the same time, the production capacity and technological level of overseas suppliers limit their ability to respond to fluctuations in demand, thereby increasing the risk of supply disruptions. Through this model, we aim to analyze and solve the key issues in the cross-border supply chain of low-carbon agricultural products, such as supply chain coordination, risk management, and revenue distribution, in order to improve the efficiency and stability of the whole supply chain. Next is the specific description and parameterization for each subject of the supply chain.

**Fig 1:**
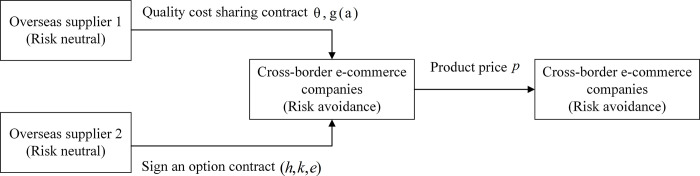
Operation mode of cross-border e-commerce import supply chain under options-quality cost sharing contract.

It is assumed that overseas supplier 1 May have supply interruption in overseas production, transportation and other links. The cross-border e-commerce import supply chain operation model under the option-quality cost-sharing contract is shown in Fig 1.

The probability of supply interruption is *β*, while the supply of overseas supplier 2 is stable and no supply interruption will occur. After the interruption occurs, cross-border e-commerce enterprises will only purchase from overseas supplier 2. In order to increase the sales volume of low-carbon agricultural products and improve consumer satisfaction, especially to meet consumers’ requirements on the quality of low-carbon agricultural products, overseas suppliers will take a series of measures to improve the quality of low-carbon agricultural products, such as strict control of product quality inspection and optimization of production process. It is assumed that there is no quality difference between the low-carbon agricultural products provided by two overseas suppliers, and the product quality level of both is *α*,*α*∈[0,+∞], The overseas suppliers need to pay the corresponding effort cost to reach the target quality level. The effort cost is the function of quality level *α*, denoting *g*(*α*). Before the beginning of the sales cycle, cross-border e-commerce first estimates the market demand and determines its order quantity *q*_*i*_ for supplier *i*, and then overseas suppliers make production plans based on this. Among them, unit cost and quality effort cost of overseas supplier are *c*_*i*_ and *g*(*a*) respectively. Unit cost *c*_*i*_ of overseas supplier is composed of unit fixed cost *c*_*fi*_ and unit variable cost *c*_*vi*_, and satisfies $c_{i}=c_{fi}+c_{vi}=\frac{c(f_{i})}{q_{i}}+c_{vi}$, *c*(*f*_*i*_) is fixed investment cost. Overseas suppliers *i* supply to cross-border e-commerce businesses at a wholesale unit price *w*_*i*_ based on costs and margins. Then, after the product sales cycle begins, cross-border e-commerce enterprises will sell low-carbon agricultural products at the price *p*. If the market demand is greater than the ordered quantity of cross-border e-commerce enterprises, overseas suppliers and cross-border e-commerce enterprises will bear the unit out-of-stock cost *v*_*s*_, *v*_*r*_ respectively. On this basis, the option contract coordination mechanism of quality cost sharing is introduced. That is, cross-border e-commerce enterprises order *h* unit product option from overseas supplier 2 in advance, and then decide whether to execute the option according to market demand, supply interruption, etc. The option ordering price and option exercise price are *k* and *e* respectively. At the same time, overseas suppliers and cross-border e-commerce enterprises bear a certain proportion of quality costs respectively. The quality cost sharing ratio of the two suppliers is *θ*, and *θ*∈[0,1]。

The basic assumption is as follows:

1. It is assumed that the random demand of low-carbon agricultural products in the whole sales cycle adopts the form of addition, the actual market demand meets *X*(*α*,*ε*) = *ηy*(*α*)+*ε*, where η is the quality effort cost coefficient affecting the demand degree, *ε* is the non-negative random variable subject to uniform distribution on [a, b], and the expectation of market random demand is *E*(*X*) = *μ*. *y*(*α*), continuous non-negative second-order differentiable, is an increasing function of the quality level *α* of low-carbon agricultural products, and is a concave function. At the same time, it is assumed that the quality effort cost function *g*(*α*) is an increasing function about the quality level of low-carbon agricultural products, and is a convex function, that is, *g*’’(*α*)>0, indicating that the effort cost of overseas suppliers is marginal increasing about *α*.
2. As the quality level of low-carbon agricultural products increases to a certain extent, the increasing trend of market demand slows down. Here, it is assumed that the market demand distribution function *F*(*x*|*α*) is a strictly decreasing function about the quality level of low-carbon agricultural products, and *f*(*x*|*α*) is a function of demand density.
3. *G*(*q*,*α*) is the expected sales volume of overseas suppliers under the quality level of low-carbon agricultural products *α*, namely $G(q,a)=E[\min(q,x)]=\int_{0}^{q}\bar{F}(x|\alpha )dx$, $\bar{F}(x|\alpha )=1-F(x|a)$。*L*(*q*,*α*) is the expected shortage quantity of low-carbon agricultural products when the quality level is *α*, namely L(q,α)=E[max(x−q)]=μ−G(q,α).
4. Let *p*>*k*+*e*>*w*_2_>*w*_1_, *c*_*i*_*q*_*i*_>*g*(*α*)>0, *w*_*i*_>*c*_*i*_>*k*>0. *p*>*k*+*e*>*w*_2_>*w*_1_, it means that the sum of the purchase price and exercise price of the option is less than the sales price of cross-border e-commerce enterprises and higher than the wholesale price of overseas suppliers, which means that cross-border e-commerce enterprises can obtain certain profits after the introduction of the option. *c*_*i*_*q*_*i*_>*g*(*α*)>0 indicates that the quality effort cost of low-carbon agricultural products after improved measures will inevitably be lower than the cost of the original low-carbon agricultural products of overseas suppliers. *w*_*i*_>*c*_*i*_>*k*>0 indicates that the option purchase price is less than the unit cost of the overseas supplier, which guarantees the validity of the option contract.

The main parameters used in this paper are shown in Table 1.

**Table 1:** Parameter description and description.

parameter	Parameter description
*w*_*i*_	Wholesale price of units sold by overseas supplier *i* to cross-border e-commerce enterprises
*p*	Unit commodity sales price of cross-border e-commerce
*v*_*s*_	Cost per unit of goods out of stock from overseas suppliers
*v*_*r*_	Unit commodity shortage cost of cross-border e-commerce
*c*_*i*_	Unit production costs of overseas suppliers
*c*_*fi*_	Unit fixed costs of overseas suppliers
*c*_*vi*_	Unit variable costs of overseas suppliers
*c*(*f*_*i*_)	Fixed investment costs of overseas suppliers
*q*_*i*_	Order quantity of cross-border e-commerce enterprises
*h*	Options for products ordered by cross-border e-commerce enterprises from overseas suppliers during the advance period
*k*	Option order price
*e*	Option strike price
*α*	Quality level of overseas suppliers
*θ*	Quality cost sharing ratio between two suppliers
*η*	A quality effort cost factor affecting the degree of demand
*ε*	A nonnegative random variable subject to uniform distribution on [a, b]

### Analysis of supply chain procurement mode under the risk aversion attitude of cross-border e-commerce

On the basis of the above model assumptions and parameter descriptions, the following low-carbon agricultural supply chain models under decentralized decision-making and centralized decision-making are constructed and verified by derivation. Then, we analyze the influence of optimal procurement quantity, supply interruption risk probability, quality level, contract parameters on the optimal decision-making of each subject from the perspective of mathematical derivation, as well as how the contract coordination is realized.

### Optimal purchase quantity based on product quality level under decentralized decision making

The expected profit or utility of the supply chain of decentralized cross-border e-commerce enterprises is analyzed based on the master-slave game theory. Both overseas suppliers and risk-averse cross-border e-commerce enterprises determine their operation strategies with their own profit or utility maximization as the goal. Two overseas suppliers determine their optimal product quality level *α*, while cross-border e-commerce enterprises with risk-averse attitude determine their optimal purchase quantity according to the market information they have acquired.

When a cross-border e-commerce enterprise holds a risk-neutral attitude, its expected profit is

$$E(\pi_{r}^{d})=\beta [pG(q_{2},\alpha )-v_{r}L(q_{2},\alpha )-w_{2}q_{2}]+(1-\beta )[pG(q_{1}+q_{2},\alpha )-v_{r}L(q_{1}+q_{2},\alpha )-w_{1}q_{1}-w_{2}q_{2}]$$ (1)

Property 1: There are four profit break-even points for risk-neutral cross-border e-commerce in supply chain under decentralized decision-making, which are as follows: $k_{1}=\frac{w_{2}q_{2}}{p}$, $k_{2}=\frac{(p-w_{2}-v_{r})q_{2}}{v_{r}}$, $k_{3}=\frac{w_{1}q_{1}+w_{2}q_{2}}{p}$, $k_{4}=\frac{\left(p+v_{r})(q_{1}+q_{2})-(w_{1}q_{1}+w_{2}q_{2}\right)}{v_{r}}$。

Proof: When the supply of overseas supplier 1 is interrupted or not interrupted, the profit of cross-border e-commerce is $\pi_{r1}^{d}$ and $\pi_{r2}^{d}$ respectively:

$$\pi_{r1}^{d}=\{\begin{array}{cc} px-w_{2}q_{2} & x<q_{2}\\ (p-w_{2})q_{2}+(q_{2}-x)v_{r} & x\geq q_{2} \end{array}$$ (2)

$$\pi_{r2}^{d}=\{\begin{array}{cc} px-wq_{1}-w_{2}q_{2} & x<q_{1}+q_{2}\\ (p+v_{r})(q_{1}+q_{2})-(w_{1}q_{1}+w_{2}q_{2})-xv_{r} & x\geq q_{1}+q_{2} \end{array}$$ (3)

1. When *x*<*q*_2_, let $\pi_{r1}^{d}=px-w_{2}q_{2}=0$, that is $k_{1}=\frac{w_{2}q_{2}}{p}$, and because $\frac{d\pi_{r1}^{d}}{dx}=p>0$, the actual market demand *x* increase with $\pi_{r1}^{d}$ strictly monotonically. If *x*<*k*_1_ then $\pi_{r1}^{d}\in (-\infty ,0)$. Conversely, if *k*_1_<*x*<*q*_2_, then $\pi_{r1}^{d}\in (0,+\infty )$. When *x*>*q*_2_, set $\pi_{r1}^{d}=(p-w_{2})q_{2}+(q_{2}-x)v_{r}=0$ to get $k_{2}=\frac{(p-w_{2}-v_{r})q_{2}}{v_{r}}$, and because $\frac{d\pi_{r1}^{d}}{dx}=-v_{r}<0$, $\pi_{r1}^{d}$ is a strictly monotone decreasing function with respect to the actual market demand. If *x*>*k*_2_, $\pi_{r1}^{d}\in (-\infty ,0)$. If *q*_2_<*x*<*k*_2_, then $\pi_{r1}^{d}\in (0,+\infty )$. In summary, $\pi_{r1}^{d}\in (-\infty ,0)$ when *x*<*k*_1_ or *x*>*k*_2_, and $\pi_{r1}^{d}\in (0,+\infty )$ when *k*_1_<*x*<*k*_2_.
2. When *x*<*q*_1_+*q*_2_, let $\pi_{r2}^{d}=px-wq_{1}-w_{2}q_{2}=0$, $k_{3}=\frac{w_{1}q_{1}+w_{2}q_{2}}{p}$ is obtained. When *x*>*q*_1_+*q*_2_, let $\pi_{r2}^{d}=(p+v_{r})(q_{1}+q_{2})-(w_{1}q_{1}+w_{2}q_{2})-xv_{r}=0$, the break-even point *k*_4_ is obtained. By the same token, if *x*<*k*_3_ or *x*>*k*_4_, we have $\pi_{r2}^{d}\in (-\infty ,0)$, and if *k*_3_<*x*<*k*_4_, we have $\pi_{r2}^{d}\in (0,+\infty )$.

Property 1 indicates that when cross-border e-commerce holds a risk-neutral attitude, no matter it orders from overseas supplier 2 or two overseas suppliers at the same time, only within a reasonable order range can cross-border e-commerce profit be guaranteed. To discuss the ordering decision under the risk aversion attitude of cross-border e-commerce, the following form of risk avoidance utility function should be introduced.


$$U(W)=\lambda (W-W_{0})$$ (4)

In the above equation, *λ* is the coefficient of risk avoidance attitude, and its value range is [1+∞). When *λ*>1, it indicates that cross-border e-commerce has a risk aversion attitude, and the higher the *λ* value, the higher the risk aversion degree. When *λ* = 1, it is a special case, indicating that cross-border e-commerce has neither risk aversion nor risk preference, and holds a risk-neutral attitude. *W* is the expected profit, and *W*_0_ is the target point of profit and loss point without loss of generality. Let *W*_0_ = 0.

By combining Eqs (2)–(4), the expected utility function of risk-averse cross-border e-commerce enterprises can be written as follows:

$$\begin{array}{l} E[U(\pi_{r}^{d})]=E(\pi_{r}^{d})+(\lambda -1)\beta [\int_{0}^{k_{1}}(px-w_{2}q_{2})f(x)dx+\int_{k_{2}}^{+\infty }\left((p-w_{2})q_{2}+v_{r}(q_{2}-x))f(x\right)dx]+\\ \left(\lambda -1)(1-\beta )[\int_{0}^{k_{3}}(px-w_{1}q_{1}-w_{2}q_{2})f(x)dx+\int_{k_{4}}^{+\infty }((p+v_{r})(q_{1}+q_{2})-(w_{1}q_{1}+w_{2}q_{2})-xv_{r})f(x)dx\right] \end{array}$$ (5)

Under ∀*α*∈(0,+∞), we take the first and second partial derivatives with respect to *q*_1_ with $E[U(\pi_{r}^{d})]$.

$$\frac{\partial E[U(\pi_{r}^{d})]}{\partial q_{1}}=(1-\beta )[(p+v_{r})\bar{F}(q_{1}+q_{2})-w_{1}]-(1-\beta )(\lambda -1)[w_{1}F(k_{3})-(p+v_{p}-w_{1})\bar{F}(k_{4})]$$ (6)

$$\frac{\partial^{2}E[U(\pi_{r}^{d})]}{\partial q_{1}^{2}}=(\beta -1)[\left(p+v_{r})f(q_{1}+q_{2}\right)]-(1-\beta )(\lambda -1)[\frac{w_{1}^{2}}{p}f(k_{3})+\frac{{\left(p+v_{r}-w_{1}\right)}^{2}}{v_{r}}f(k_{4})]$$ (7)

Since $p-w_{1}>0,v_{r}>0,\lambda >1,\beta <1$, we can see that $\frac{\partial^{2}E[U(\pi_{r}^{d})]}{\partial q_{1}^{2}}<0$, so the expected utility function $E[U(\pi_{r}^{d})]$ of cross-border e-commerce is strictly concave function about *q*_1_. Similarly, we can get $\frac{\partial^{2}E[U(\pi_{r}^{d})]}{\partial q_{1}^{2}}<0$, that is, there is a unique optimal order quantity $q_{1}^{d^{*}}$, $q_{2}^{d^{*}}$ satisfies the equation:

$$[(p+v_{r})\bar{F}(q_{1}^{d^{*}}+q_{2}^{d^{*}})-w_{1}]-(\lambda -1)[w_{1}F(k_{3})-(p+v_{r}-w_{1})\bar{F}(k_{4})]=0$$ (8)

$$\begin{array}{l} \beta (p+v_{r})\bar{F}(q_{2}^{d^{*}})+(1-\beta )(p+v_{r})\bar{F}(q_{1}^{d^{*}}+q_{2}^{d^{*}})-w_{2}-(\lambda -1)[\beta (w_{2}F(k_{1})-\\ (p+v_{r}-w_{2})\bar{F}(k_{2}))-(1-\beta )(w_{2}F(k_{3})-(p+v_{r}-w_{2})\bar{F}(k_{4}))]=0 \end{array}$$ (9)

The total expected profit function of the two overseas suppliers is:

$$\begin{array}{l} E(\pi_{s}^{d})=(1-\beta )[\left(w_{1}-c_{1})q_{1}-2v_{s}L(q_{1}+q_{2},\alpha )-g(\alpha \right)]+\\ \beta [\left(w_{2}-c_{2})q_{2}-g(\alpha )-v_{s}L(q_{2},\alpha \right)] \end{array}$$ (10)

For *∀α*∈(0,+*∞*), $E(\pi_{s}^{d})$ is a concave function of *α*, and there is a unique optimal product quality level where $\alpha^{d^{*}}$ satisfies:

$$(1-\beta )[2v_{s}\int_{0}^{q_{1}+q_{2}}\frac{\partial F(x|\alpha )}{\partial \alpha }dx+g^{\prime }(\alpha )]+\beta [v_{s}\int_{0}^{q_{2}}\frac{\partial F(x|\alpha )}{\partial \alpha }dx+g^{\prime }(\alpha )]=0$$ (11)

Theorem 1: Under the decentralized decision-making mode, the product quality level *α*(*q*_1_,*q*_2_) of overseas suppliers will increase with the increase of cross-border e-commerce enterprises’ purchasing quantity, while the cross-border e-commerce enterprises’ purchasing quantity *q*_*i*_(*α*) will also increase with the improvement of overseas suppliers’ quality level.

Proof: Use the implicit function theorem to find the partial derivative of *α*(*q*_1_,*q*_2_)and *q*_*i*_(*α*) with respect to *q*_1_ and *α* respectively.


$$\frac{\partial \alpha (q_{1},q_{2})}{\partial q_{1}}=-\frac{\frac{\partial^{2}E(\pi_{s}^{d})}{\partial \alpha \partial q_{1}}}{\frac{\partial^{2}E(\pi_{s}^{d})}{\partial \alpha^{2}}}=-\frac{2(\beta -1)v_{s}\frac{\partial F(q_{1}+q_{2}|\alpha )}{\partial \alpha }}{\frac{\partial^{2}E(\pi_{s}^{d})}{\partial \alpha^{2}}}$$ (12)

$$\frac{\partial q_{1}(\alpha )}{\partial \alpha }=-\frac{\frac{\partial^{2}E[U(\pi_{r}^{d})]}{\partial q_{1}\partial \alpha }}{\frac{\partial^{2}E[U(\pi_{r}^{d})]}{\partial q_{1}^{2}}}=-\frac{\left(\beta -1)[\left(p+v_{r})\frac{\partial F(q_{1}+q_{2})}{\partial \alpha }+(\lambda -1)(w_{1}\frac{\partial F(k_{3})}{\partial \alpha }+(p+v_{r}-w_{1})\frac{\partial F(k_{4})}{\partial \alpha }\right)\right]}{\frac{\partial^{2}E[U(\pi_{r}^{d})]}{\partial q_{1}^{2}}}$$ (13)

Given that $\frac{\partial F(x|\alpha )}{\partial \alpha }<0$, $\frac{\partial^{2}E(\pi_{s}^{d})}{\partial \alpha^{2}}<0$, we can infer that $\frac{\partial^{2}E(\pi_{s}^{d})}{\partial \alpha \partial q_{1}}>0$, that is $\frac{\partial \alpha (q_{1},q_{2})}{\partial q_{1}}$. Meanwhile, it can be obtained from Eq (7) that $\frac{\partial^{2}E[U(\pi_{r}^{d})]}{\partial q_{1}^{2}}<0$, and $\frac{\partial^{2}E[U(\pi_{r}^{d})]}{\partial q_{1}\partial \alpha }>0$, that is $\frac{\partial q_{1}(\alpha )}{\partial \alpha }>0$. Similarly, the correlation between *α*(*q*_1_,*q*_2_), *q*_2_(*α*) and *q*_2_,*α* can be obtained. The product quality level of overseas suppliers *α*(*q*_1_,*q*_2_) and cross-border e-commerce purchase quantity *q*_1_ and *q*_2_ are mutually increasing. Theorem 1 states that with the increase of cross-border e-commerce purchase volume, overseas suppliers will be prompted to improve product quality in terms of technology and function. Meanwhile, the improvement of quality level will meet consumers’ requirements for product quality and increase product sales.

#### The optimal purchase quantity based on product quality level under centralized decision

In the case of centralized decision-making, two overseas suppliers are regarded as upstream supply departments of cross-border e-commerce enterprises, and the overall maximum profit or utility is obtained through cooperative game. It is assumed that all members hold a risk-neutral attitude and make a unified decision to determine the risk strategy to deal with the problem of cross-border supply chain disruption, that is, to select the optimal product quality level and optimal purchase volume to achieve the profit target. At this time, the expected profit function of the cross-border e-commerce supply chain is:

$$\begin{array}{l} E(\pi_{s}^{c})=(1-\beta )[pG(q_{1}+q_{2},\alpha )-(v_{r}+2v_{s})L(q_{1}+q_{2},\alpha )-c_{1}q_{1}-c_{2}q_{2}-2g(\alpha )]+\\ +\beta [pG(q_{2},\alpha )-(v_{r}+v_{s})L(q_{2},\alpha )-c_{2}q_{2}-g(\alpha )] \end{array}$$ (14)

Take the partial derivative with respect to *q*_1_ and *q*_2_, respectively:

$$\frac{\partial E(\pi_{s}^{c})}{\partial q_{1}}=(1-\beta )[(p+2v_{s}+v_{r})\bar{F}(q_{1}+q_{2})-c_{1}]$$ (15)

$$\frac{\partial E(\pi_{s}^{c})}{\partial q_{2}}=(1-\beta )[(p+2v_{s}+v_{r})\bar{F}(q_{1}+q_{2})-c_{2}]+\beta (p+v_{s}+v_{s})\bar{F}(q_{2})-c_{2}]$$ (16)

The Hess matrix of the cross-border supply chain is further calculated and analyzed as negative definite, and the profit function of the cross-border supply chain has the maximum value. The equations are obtained by linking (15) and (16), so that the optimal purchase quantity $q_{1}^{c^{*}}$, $q_{2}^{c^{*}}$ of cross-border e-commerce meet the following conditions:

$$\bar{F}(q_{2}^{c^{*}})=\frac{c_{2}+(\beta -1)c_{1}}{\beta (p+v_{s}+v_{r})}\;\bar{F}(q_{1}^{c^{⋆}}+q_{2}^{c^{⋆}})=\frac{c_{1}}{p+2v_{s}+v_{r}}$$ (17)

Calculate the partial derivative of Eq (14) with respect to *α*, and obtain the optimal product quality leve $\alpha^{c^{*}}$, which meets the following conditions:

$$(1-\beta )[(p+v_{r}+2v_{s})\int_{0}^{q_{1}+q_{2}}\frac{\partial (F(x|\alpha ))}{\partial \alpha }dx+g^{\prime }(\alpha )]+\beta [(p+v_{r}+v_{s})\int_{0}^{q_{2}}\frac{\partial (F(x|\alpha ))}{\partial \alpha }dx+g'(\alpha )]=0$$ (18)

Theorem 2: Under the centralized decision, the reaction function *α*(*q*_1_,*q*_2_) of the product quality level of overseas suppliers is a strictly increasing function of the purchase quantity of cross-border e-commerce from different channels *q*_1_,*q*_2_. The optimal response function *q*_*i*_(*α*) of cross-border e-commerce purchase volume is a strictly increasing function of the quality level *α* of overseas suppliers’ low-carbon agricultural products.

The proof of Theorem 2 is similar to the implicit function proof of Theorem 1. Theorem 2 shows that centralized decision-making is the same as decentralized decision-making, and cross-border e-commerce enterprises’ purchasing quantity and overseas suppliers’ quality level of low-carbon agricultural products are positively correlated with each other.

Theorem 3: When supply interruption occurs, the two-level supply chain based on risk-averse cross-border e-commerce and low-carbon agricultural product quality level of suppliers cannot reach the centralized decision-making level under decentralized decision-making, that is, the coordination of cross-border supply chain cannot be realized.

Proof: If the profit of cross-border supply chain under decentralized decision-making is to reach the level of profit under centralized decision-making, it should be satisfied: $q_{i}^{d^{*}}=q_{i}^{c^{*}}$, $a^{d^{*}}=a^{c^{*}}$, let $a^{d^{*}}=a^{c^{*}}$ be established, and according to Eqs (11) and (18), we can get:

$$\begin{array}{l} (1-\beta )[2v_{s}\int_{0}^{q_{1}+q_{2}}\frac{\partial F(x|\alpha )}{\partial \alpha }dx+g^{\prime }(\alpha )]+\beta [g^{\prime }(\alpha )+v_{s}\int_{0}^{q_{2}}\frac{\partial F(x|\alpha )}{\partial \alpha }dx]=\\ \left(1-\beta )[\left(p+v_{r}+2v_{s})\int_{0}^{q_{1}+q_{2}}\frac{\partial (F(x|\alpha )}{\partial \alpha }dx+g^{\prime }(\alpha \right)]+\beta [\left(p+v_{r}+v_{s})\int_{0}^{q_{2}}\frac{\partial (F(x|\alpha )}{\partial \alpha }dx+g'(\alpha \right)\right] \end{array}$$ (19)

Given the conditions *p*+*v*_*r*_>0, that is, *p*+*v*_*r*_≠0 is constant. Therefore, the counter-proof hypothesis is not valid, that is, the profit level of cross-border supply chain with decentralized decision-making cannot achieve the profit level under centralized conditions.

### Contract coordination of cross-border e-commerce supply chain with quality cost sharing

Through the above analysis, it is found that under decentralized decision-making, cross-border e-commerce and the two overseas suppliers only start from the perspective of their own interests, and cannot always achieve the optimal strategy and profit level under the centralized mode, that is, the cross-border e-commerce secondary supply chain considering the supply interruption and the quality level of low-carbon agricultural products cannot achieve coordination under simple wholesale price contract. New contracts need to be designed to coordinate this supply chain. Here, combining the idea of option contract and cost sharing, the options-quality cost sharing contract is designed to share part of the cost of quality effort of overseas suppliers by risk-averse cross-border e-commerce enterprises. Considering that risk-averse cross-border e-commerce and risk-neutral overseas suppliers share the cost of quality effort, the cost sharing coefficient is set as *θ*, that is, the quality cost function borne by cross-border e-commerce is θ*g*(*α*), and the quality cost function of overseas suppliers is(1−*θ*)*g*(*α*).

Under the new options-quality cost-sharing contract, considering the probability of supply interruption of overseas supplier 1, the expected revenue function of cross-border e-commerce is:

$$\begin{array}{c} E(\pi_{r}^{\theta })=\left(1-\beta )[pG(h+q_{1},\alpha )-q_{1}w_{1}-hk-v_{r}L(h,\alpha )-eS(h+q_{1},\alpha )-2\theta g(\alpha )\right]\\ +\beta [\left(p-e)G(h,\alpha )-hk-v_{r}L(h,\alpha )-\theta g(\alpha \right)] \end{array}$$ (20)

Among them: $S(h+q_{1},\alpha )=\int_{q_{1}}^{h+q_{1}}(x-q_{1})f(x)dx+\int_{h+q_{1}}^{+\infty }hf(x)dx$

According to property 1, the break-even points of cross-border e-commerce with a risk-neutral attitude under options-quality cost-sharing contract are respectively:

$k_{1}=\frac{kh+\theta g(\alpha )}{p-e}$, $k_{2}=\frac{\left(p-k-e+v_{r})h-\theta g(\alpha \right)}{v_{r}}$, $k_{3}=\frac{w_{1}q_{1}+kh+2\theta g(\alpha )}{p}$, $k_{4}=\frac{\left(p-w_{1}+v_{r})q_{1}+(p-k-e+v_{r})h-2\theta g(\alpha \right)}{v_{r}}$ Thus, the expected utility function of cross-border e-commerce under the risk aversion attitude can be obtained as follows:

$$\begin{array}{l} E[U(\pi_{r}^{o})]=E(\pi_{r}^{o})+(\lambda -1)[\beta \int_{0}^{k_{1}}[\left(p-e)x-kh-\theta g(\alpha \right)]f(x)dx]+\\ \beta \int_{k_{1}}^{+\infty }[\left(p-k-e)-v_{r}(x-h)-\theta g(\alpha \right)]f(x)dx+(1-\beta )\int_{0}^{k_{3}}[px-w_{1}q_{1}-kh-2\theta g(\alpha )]f(x)dx\\ +(1-\beta )\int_{k_{4}}^{+\infty }[\left(p-w_{1})q_{1}+(p-k-e)h-v_{r}(x-q_{1}-h)-2\theta g(\alpha \right)]f(x)dk] \end{array}$$ (21)

Under the condition of ∀*α*∈(0,+∞), $E[U(\pi_{r}^{o})]$ the first and second partial derivatives of *q*_1_ and *h* are obtained respectively, and the Hess matrix is judged to be negative definite, that is, the expected utility function $E[U(\pi_{r}^{o})]$ is a concave function of the purchase quantity *q*_1_ from overseas supplier 1 and the option order quantity *h* from overseas supplier 2, indicating that there is a unique optimal purchase quantity $q_{1}^{o^{*}}$, $h^{o^{*}}$, which satisfies the following equation:

$$-eF(q_{1})-(p-e-v_{r})F(q_{1}+h)+p-w_{1}+v_{r}+(1-\lambda )[w_{1}F(k_{3})-(p-w_{1}+v_{r})\bar{F}(k_{4})]=0$$ (22)

$$\begin{array}{c} -\beta (p-e+v_{r})F(h)-(1-\beta )(p-e+v_{r})F(q_{1}+h)+p-k-e+v_{r}+\beta (1-\lambda )[kF(k_{1})\\ -(p-k-e+v_{r})\bar{F}(k_{2})]+(1-\beta )(1-\lambda )[kF(k_{3})-(p-k-e+v_{r})\bar{F}(k_{4})]=0 \end{array}$$ (23)

Also, the total expected return function of the two risk-neutral overseas suppliers at this time is:

$$\begin{array}{l} E(\pi_{s}^{o})=(1-\beta )[\left(w_{1}-c_{1})q_{1}+eS(q_{1}+h,\alpha )-2v_{s}L(q_{1}+h_{2},\alpha )-(1-\theta )g(\alpha \right)]+\\ \beta [eG(h,\alpha )-v_{s}L(h,\alpha )+(k-c_{2})h-(1-\theta )g(\alpha )] \end{array}$$ (24)

For∀*α*∈(0,+∞), $E(\pi_{s}^{o})$ is a concave function of *α*, indicating that there is a unique optimal product quality level $\alpha^{o^{*}}$, satisfying the following equation:

$$\begin{array}{l} (1-\beta )[e\int_{q_{1}}^{q_{1}+h}\frac{\partial (F(x|\alpha )}{\partial \alpha }dx+2v_{s}\int_{0}^{q_{1}+h}\frac{\partial (F(x|\alpha ))}{\partial \alpha }dx+(1-\theta )g^{\prime }(\alpha )]+\\ \beta [\left(e+v_{s})\int_{0}^{h}\frac{\partial (F(x|\alpha ))}{\partial \alpha }dx+(1-\theta )g^{\prime }(\alpha \right)]=0 \end{array}$$ (25)

Theorem 4: Based on options-quality cost-sharing contract, a cross-border supply chain consisting of two risk-neutral overseas suppliers and risk-averse cross-border e-commerce can achieve supply chain coordination under the condition of reducing the probability of supply interruption and improving product quality.

Proof: To introduce product quality cost sharing into option contract and realize the coordination of the whole supply chain is to make the optimal purchase quantity of cross-border e-commerce and the optimal product quality level of overseas suppliers meet the optimal strategy under centralized decision-making.

That is $q_{1}^{c^{*}}=q_{1}^{o^{*}}$, $q_{2}^{c^{*}}=h^{o^{*}}$, $a^{c^{*}}=a^{o^{*}}$ joint vertical (17), (18), (22), (23), (25) can be obtained:

$$\left\{ \begin{array}{l} \theta =\frac{(1-\beta )e\int_{q_{1}^{o^{*}}}^{q_{1}^{o^{*}}+h^{o^{*}}}\frac{\partial (F(x|\alpha^{o^{*}})}{\partial \alpha^{o^{*}}}dx+\beta (e-p-v_{r})\int_{0}^{h^{o^{*}}}\frac{\partial (F(x|\alpha^{o^{*}}))}{\partial \alpha^{o^{*}}}dx}{g'(\alpha^{o^{*}})}-\\ \frac{(1-\beta )(p+v_{r})\int_{q_{1}^{o^{*}}}^{q_{1}^{c^{*}}+q_{2}^{c^{*}}}\frac{\partial (F(x|\alpha^{c^{*}})}{\partial \alpha^{c^{*}}}dx}{g'(\alpha^{o^{*}})}\\ F(q_{1}^{c^{*}}+q_{2}^{c^{*}}|\alpha^{c^{*}})=F(q_{1}^{o^{*}}+h^{o^{*}}|\alpha^{o^{*}})=1-\frac{c_{1}}{p+2v_{s}+v_{r}}\\ F(q_{2}^{c^{*}}|\alpha^{c^{*}})=F(h^{o^{*}}|\alpha^{o^{*}})=1-\frac{c_{2}+(\beta -1)c_{1}}{\beta (p+v_{s}+v_{r})} \end{array}\right.$$ (26)

As shown in the above equation, the supply chain can be optimized and coordinated only when the purchase volume, sharing coefficient and product quality level of cross-border e-commerce meet the above conditions.

## Results and discussion

When the supply chain of cross-border e-commerce considers the risk of supply interruption and product quality control, there is a game among the participants. The optimal product quality level of overseas suppliers and the optimal purchase quantity of cross-border e-commerce depend on the selection of relevant parameters of the model. The above models are analyzed with examples, and the optimal strategies under different decision-making modes are compared. The influences of supply interruption risk probability, risk avoidance coefficient, product quality level and other parameters on the profit and utility of cross-border supply chain are analyzed, so as to verify whether options-quality cost-sharing contract can achieve supply chain coordination. In order to facilitate calculation, it is assumed that the market demand function is $F(X)=\eta \sqrt{\alpha }+\varepsilon$, the market random demand variable *ε* follows the uniform distribution on the interval [1,100], and the product quality input costs of overseas suppliers are all $g(\alpha )=\frac{\mu \alpha^{2}}{2}=25\alpha^{2}$. According to the objective realistic environment, other relevant parameters of the model are set as follows: *p* = 90, *w*_1_ = 60, *w*_2_ = 65, *c*_1_ = 20, *c*_2_ = 25, *v*_*s*_ = 2, *v*_*r*_ = 4, *λ* = 2, *η* = 2.

### Comparison of optimal purchasing strategies under different operation modes

**Fig 2:**
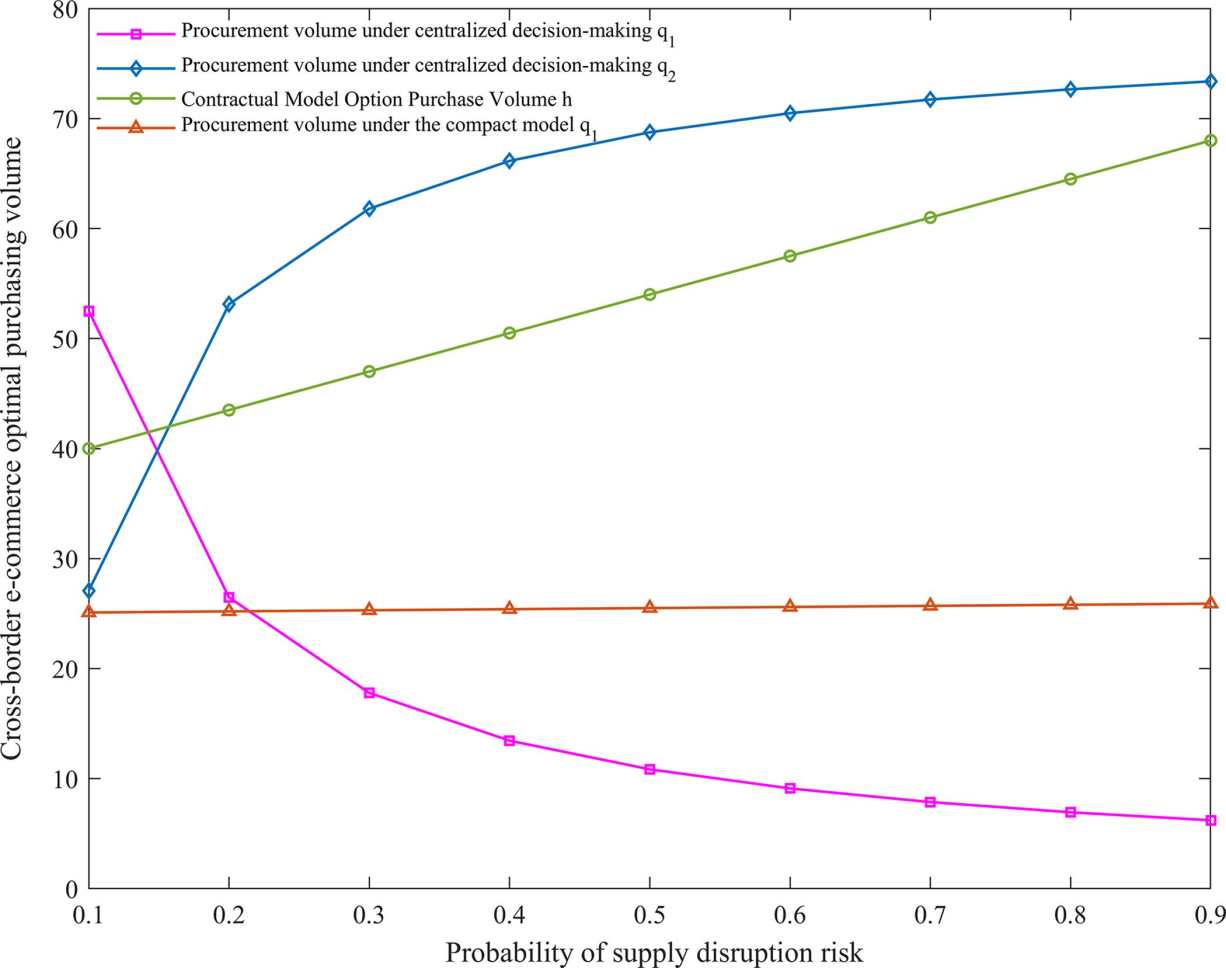
*β* influence on optimal purchasing volume of cross-border e-commerce.

After the centralized decision-making mode and the introduction of options-quality sharing contract, the change of supply interruption risk probability on the optimal purchase volume of retailers can be judged whether the improved contract model can enhance the supply stability of cross-border e-commerce supply chain and meet the market demand, as shown in Fig 2.

Our numerical simulation results (Fig 2) show that as the risk of supply disruption *β* increases, cross-border e-commerce firms reduce their purchases from the riskier overseas supplier 1 and increase their purchases from the less risky overseas supplier 2. In contrast to existing studies, we further note that this variation in purchases is particularly pronounced in the interval (0,0.3) where the risk of supply disruption is low. Provides a new perspective for understanding the dynamic adjustment of cross-border supply chains of low-carbon agricultural products under different levels of risk. In addition, we find that despite the increased risk of supply disruptions from overseas supplier 1, the total expected profits of both suppliers are less sensitive to the risk of disruptions. This suggests that the risk tolerance of supply chains may vary depending on the structure and mode of cooperation, a finding that contrasts with the results of some studies that emphasize that supply chains are highly sensitive to risk [26]. Under the condition of introducing the option-quality cost-sharing contract, the number of product options ordered by cross-border e-commerce enterprises from overseas supplier 2 increases with *β*. However, the quantity of products purchased by overseas supplier 1 does not obviously remain stable with *β*, and the difference between the probability of risk *β* enhancement and *q*_1_ also keeps increasing. This point complements the existing discussion in the literature on risk management strategies for cross-border supply chains of low-carbon agricultural products and emphasizes the importance of option contracts in risk aversion.

### The influence of different contract parameters on the expected return of cross-border e-commerce enterprises’ supply chain

According to Theorem 3, the supply chain composed of risk-neutral overseas suppliers and risk-averse cross-border e-commerce cannot achieve overall coordination under decentralized decision-making, that is, it cannot reach the optimal product quality level and optimal purchase quantity under centralized decision-making. Therefore, the influence of various parameters on the expected profit of cross-border supply chain under decentralized conditions is not discussed here.

1. Change of expected profit of cross-border supply chain under centralized decision-making

**Fig 3:**
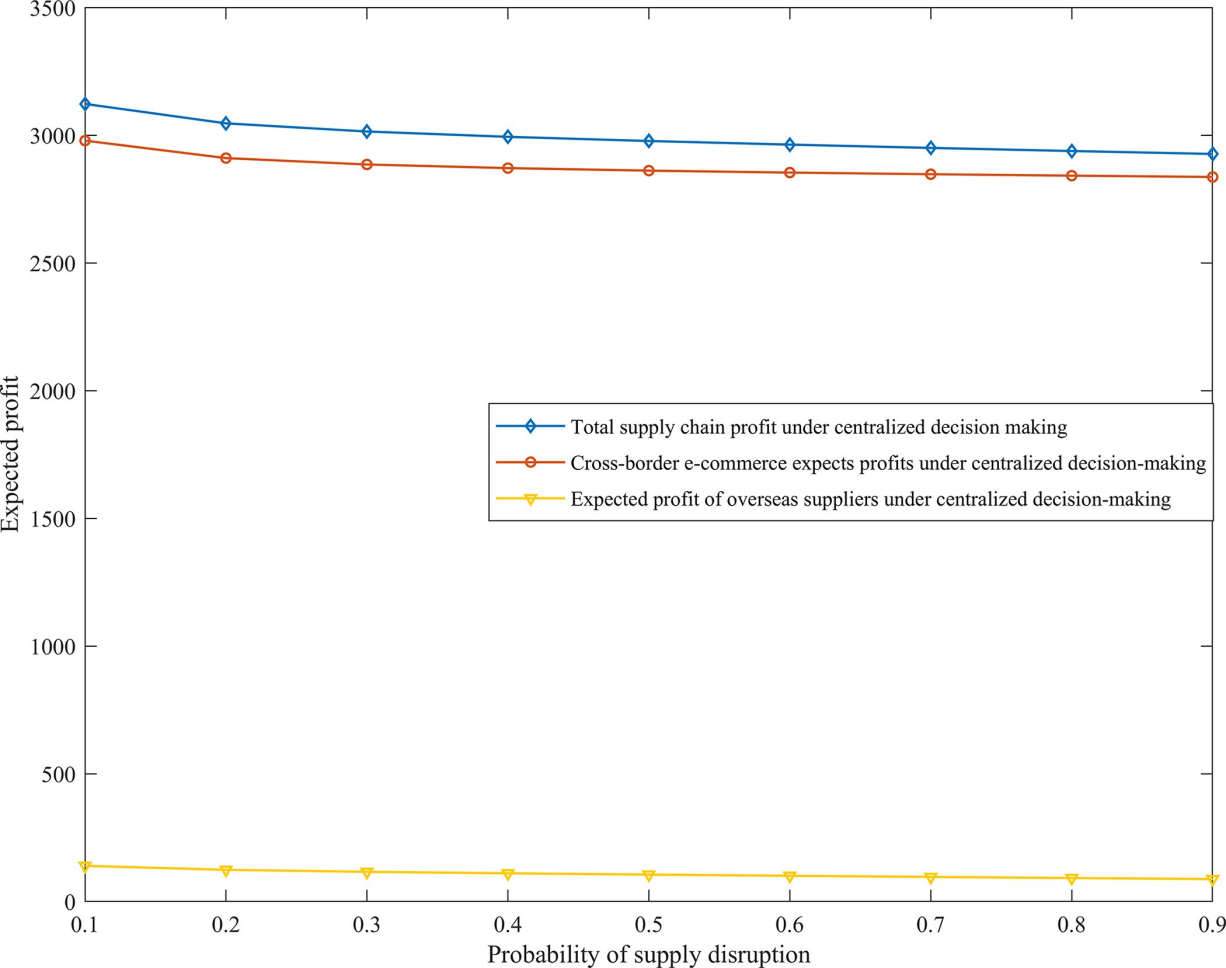
The effect of supply disruption probability *β* on expected profit of cross-border supply chain under centralized decision-making.

**Fig 4:**
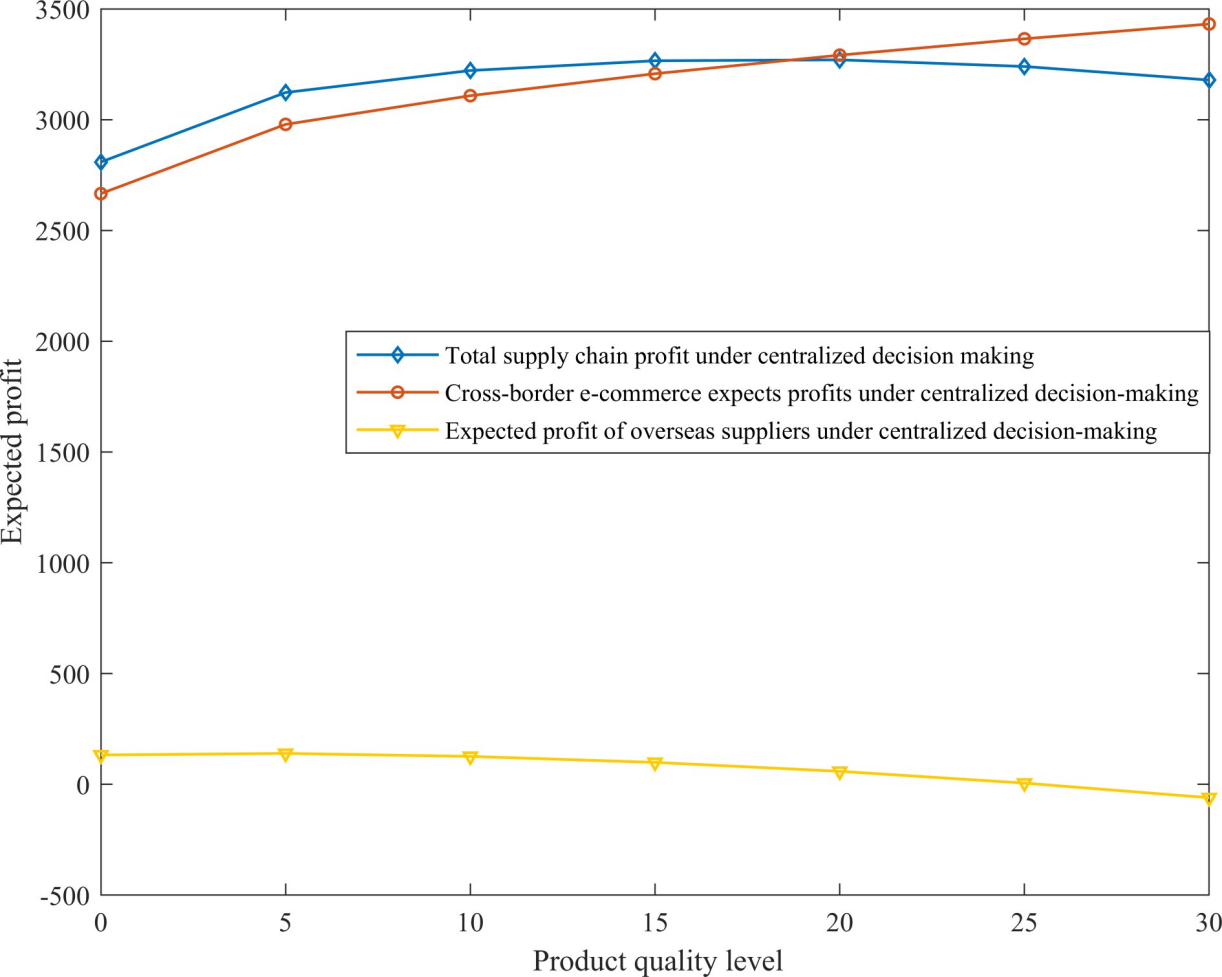
The effect of product quality level *α* on the expected profit of cross-border supply chain under centralized decision-making.

Assuming that product quality level *α* = 5 and supply interruption probability *β* = (0.1:0.9), the variation trend diagram of different supply interruption risk probabilities and the expected profits of cross-border e-commerce and overseas suppliers is obtained, as shown in Fig 3. Assume that the risk interruption probability *β* = 0.3, and the supplier’s product quality effort cost should be less than the supplier’s production cost. Set product quality level *α* = (5,30) here, and the variation trend chart of different product quality levels and the expected profits of cross-border e-commerce and overseas suppliers can be obtained, as shown in Fig 4.

Numerical simulation results show that supply disruption probability *β* and product quality level *α* are two key factors affecting the expected profit of cross-border supply chains. Fig 3 shows that as the risk of supply disruption from overseas supplier 1 increases, the expected profit of cross-border e-commerce decreases accordingly, consistent with the findings of existing studies. However, our study further points out that the total expected profit of the two suppliers is less sensitive to the risk of disruption and does not change significantly, suggesting that the supply chain’s resilience to risk may vary depending on the structure and mode of cooperation. Our results show that under centralized decision-making, cross-border e-commerce companies and overseas suppliers are able to maximize the overall profit of the system through in-depth cooperation or alliances, where the expected profit of cross-border e-commerce companies is much higher than that of overseas suppliers, which has not been sufficiently emphasized in the existing literature. As can be seen in Fig 4, unlike the results of some studies that suggest that product quality positively affects profits [27], our study reveals that as product quality improves, overseas suppliers’ expected profits decrease due to increased costs, suggesting that excessively high product quality levels are not always conducive to profit growth. At the same time, cross-border e-commerce firms are favored by consumers due to improved product quality, the share of market demand expands, and their expected profits continue to rise, especially when the product quality level is high, the rise in profits is more significant, which complements the discussion of the drivers of cross-border e-commerce profit growth in existing studies. Thus, this study proposes that in order to ensure the smooth operation of the supply chain and maximize the overall profit, cross-border e-commerce enterprises not only need to take measures to reduce the risk of supply disruptions, but also need to cooperate with overseas suppliers so that the supply chain maintains its operation in the interval of low risk of supply disruptions and reasonable level of product quality. This perspective emphasizes the importance of supply chain coordination in risk management and profit maximization and provides new strategies for supply chain management practices.

(2) Expected profit change of cross-border supply chain under options-quality cost-sharing contract

**Fig 5:**
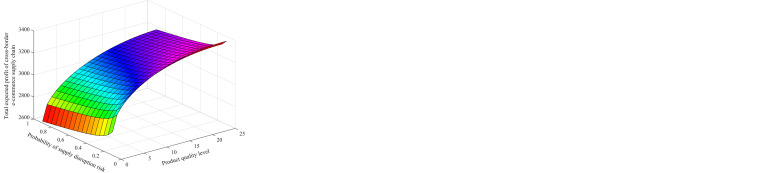
The effect of contract parameters *α*,*β* on the expected profit of cross-border supply chain, (a) Change in expected profit for two overseas suppliers in aggregate, (b) Change in expected profit for cross-border e-commerce firms, (c) Change in expected profit of the cross-border e-commerce firm’s supply chain as a whole.

Assume that the supply interruption risk probability *β* = (0.1,0.9), and adjust the value range of product quality level *α* to (0, 25) according to the above, the variation trend chart of different supply interruption risk probability and product quality level with cross-border e-commerce, two overseas suppliers and the overall expected profit of cross-border e-commerce supply under the contract mode is obtained, as shown in Fig 5.

Based on the simulation results Fig 5, we conclude that under the option-quality cost-sharing contract model, the expected profit of cross-border e-commerce enterprises shows a decreasing trend as the probability of supply disruption risk of overseas supplier 1 increases. At the same time, the overall expected profits of the two overseas suppliers showed an upward trend. This suggests that, faced with the risk of supply disruption, cross-border e-commerce firms tend to hedge their risks by entering into option contracts. However, when the risk of supply disruption is too high, the option price of overseas supplier 2 rises, which may squeeze the profit margins of cross-border e-commerce firms while increasing the availability and expected profits of overseas supplier 2. In addition, higher product quality levels have a positive impact on the expected profitability of cross-border e-commerce firms, the two overseas suppliers, and the cross-border supply chain as a whole, a trend that is similar to the changes under centralized decision-making. By combining the analysis in Figs 3 and 5(C), we can find that the overall expected profit of the cross-border supply chain under the contractual model can reach the same level as under the centralized decision. This finding again verifies Theorem 4 that improved contracting can achieve supply chain coordination.

**Fig 6:**
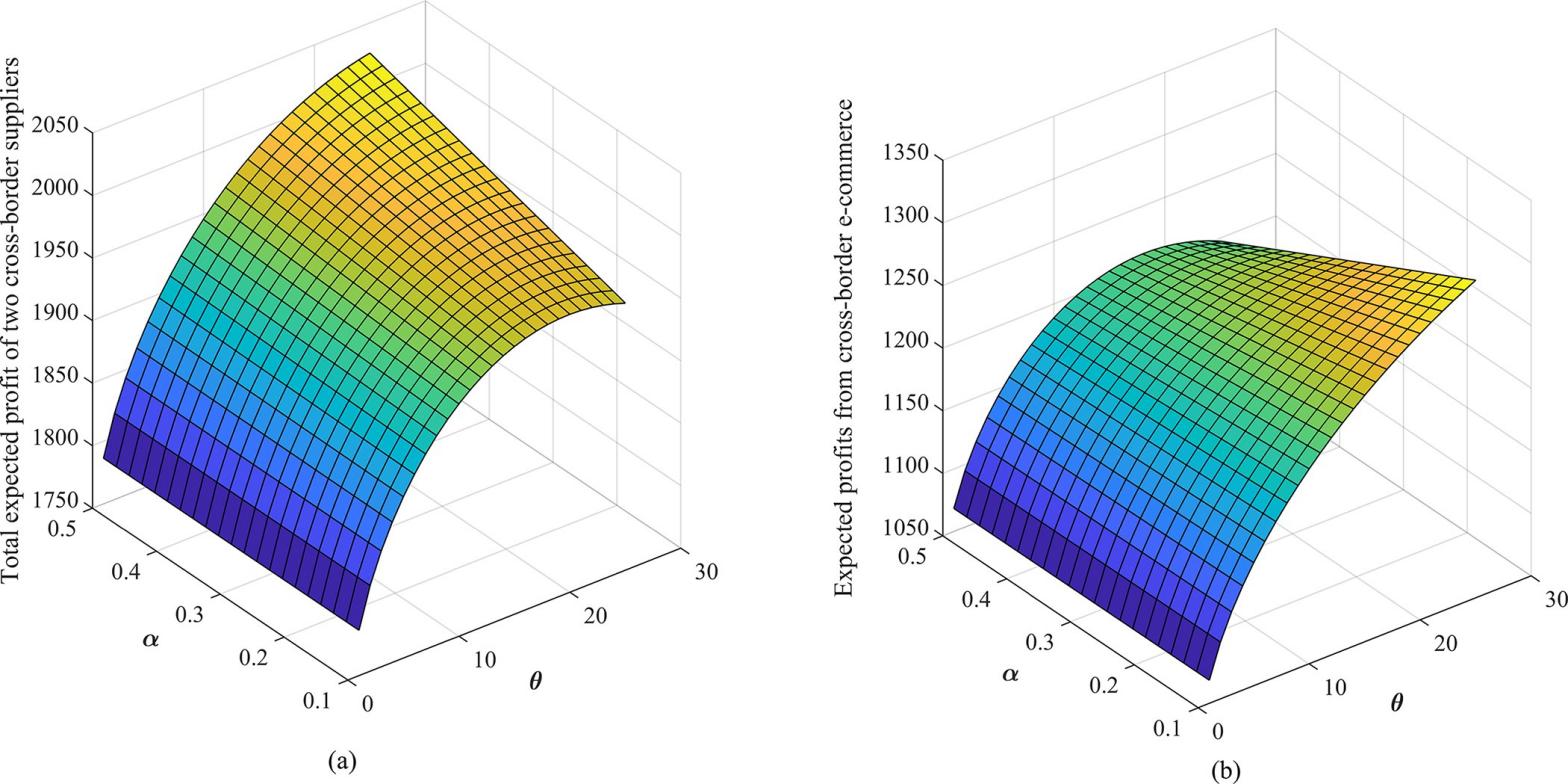
The effect of contract parameters *θ*,*α* on the expected profit of cross-border supply chain: (a) expected profit of overseas suppliers, (b) cross-border e-commerce expected profits.

**Fig 7:**
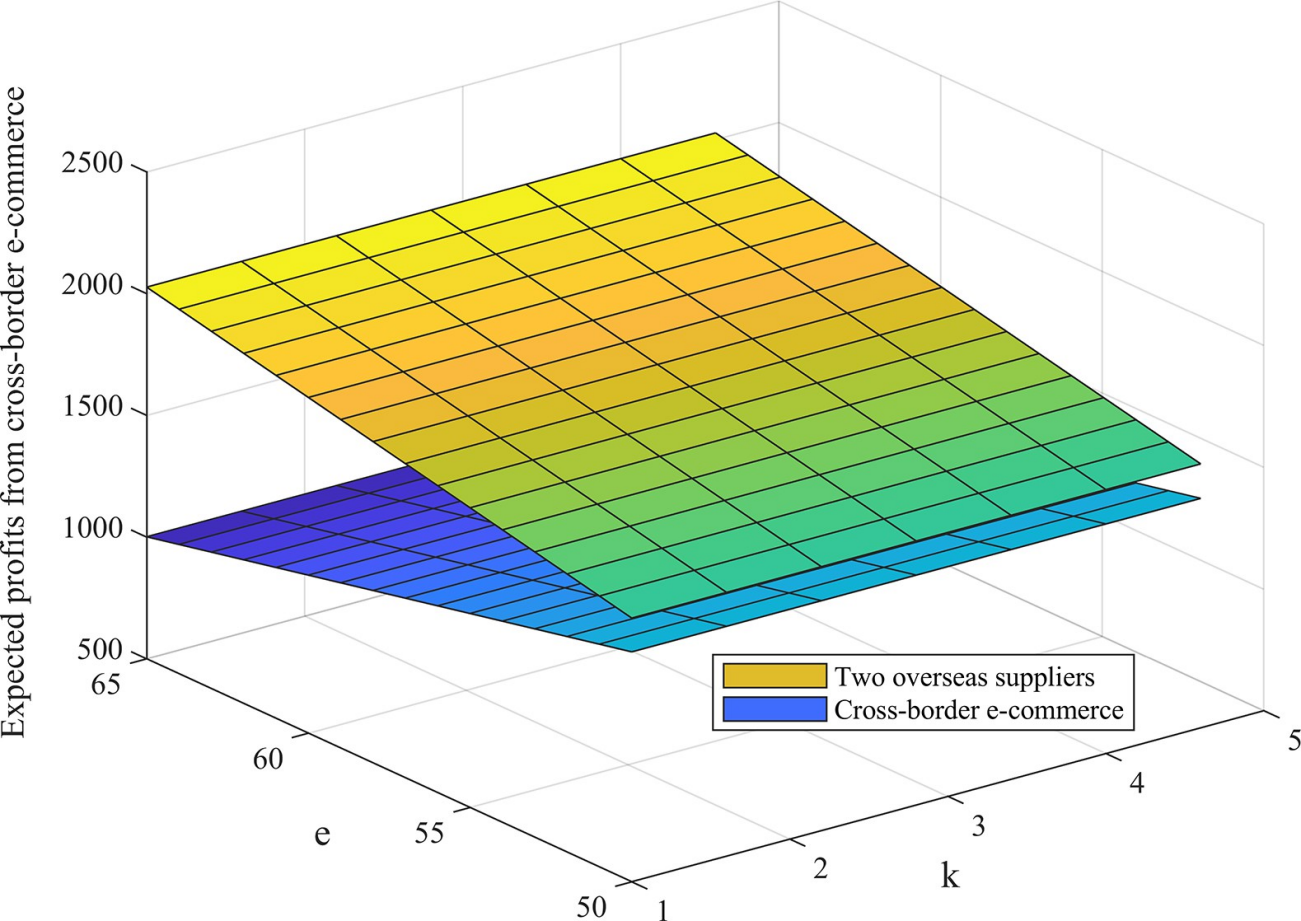
The effect of contract parameters *k*,*e* on the expected profit of cross-border supply chain.

The following is a discussion of the effects of cost sharing coefficient *θ*, option ordering price *k*, option exercise price *e* and other parameters in the option-quality cost sharing model on the expected profit of cross-border supply chain under different combination conditions, as shown in Figs 6 and 7.

Fig 6 shows that the cost sharing coefficient is positively correlated with the expected profit of cross-border e-commerce firms and negatively correlated with the expected profit of overseas suppliers, which is consistent with some studies that emphasize the role of cost sharing in supply chain profit distribution. It is further concluded from our study that the impact sensitivity of the cost sharing coefficient is lower than the product quality level, which indicates that product quality control is equally important when considering cost sharing strategies. Our study finds that there exists an optimal cost-sharing coefficient that maximizes the respective revenues of cross-border e-commerce firms and overseas suppliers. This view provides a new perspective on supply chain coordination and emphasizes the importance of finding optimal cost-sharing points in supply chain design. As can be seen from Fig 7, an increase in the option subscription price and strike price leads to an increase in the expected return of the overseas supplier 2 and a decrease in the expected return of the cross-border e-commerce provider. This finding complements the discussion of the impact of option prices on supply chain profit sharing in a number of studies, but in particular we emphasize the higher degree to which option strike prices affect the expected profits of supply chain members. And we observe that different combinations of contract parameters lead to different profit allocation scenarios between cross-border e-commerce firms and overseas retailers, further emphasizing the role of firms’ operational strength and bargaining power in profit allocation.

## Conclusion

In this paper, the supply interruption risk and product quality control of cross-border import supply chain are considered, and a cross-border e-commerce secondary supply chain consisting of risk-averse cross-border e-commerce enterprises and two risk-neutral overseas suppliers is constructed. The optimal decision-making and supply chain coordination of cross-border e-commerce and overseas suppliers under centralized and decentralized decision-making are respectively compared. On this basis, the option contract model is constructed by integrating the idea of quality cost sharing, and the impact of different parameters on the supply chain system is analyzed through arithmetic examples. Through the study, it is found that:

1. Under centralized decision making, cross-border e-commerce firms reduce purchases from riskier suppliers and shift to less risky suppliers when the risk of supply disruption increases.
2. Rising supply disruption risk leads to lower profits for cross-border e-commerce firms, and improved product quality leads to higher costs for dominant overseas suppliers, reducing profits for dominant overseas suppliers.
3. Under the contractual model, cross-border e-commerce firms’ profits fall while overseas suppliers’ profits rise when the risk of supply disruptions increases; improved product quality generally increases all parties’ profits.
4. The cost-sharing coefficient positively affects the profit of cross-border e-commerce enterprises and negatively affects the profit of overseas suppliers. When the option ordering price and execution price increase, the expected returns of overseas suppliers2 will gradually increase, and on the contrary the expected returns of cross-border e-commerce companies gradually decrease.

## Supporting information

S1 Appendix Relevant simulation results and code data are in the folder S1 Appendix. (ZIP)

S1 Data Simulation parameters are set in the file S1 Data. (DOCX)
