# Supplementary figures and images for: Cross-border supply chain coordination of low-carbon agricultural products under the risk of supply uncertainty
Source: PLoS One. 2024 Oct 22;19(10):e0309763. doi: 10.1371/journal.pone.0309763 (PMC11495562; doi:10.1371/journal.pone.0309763)

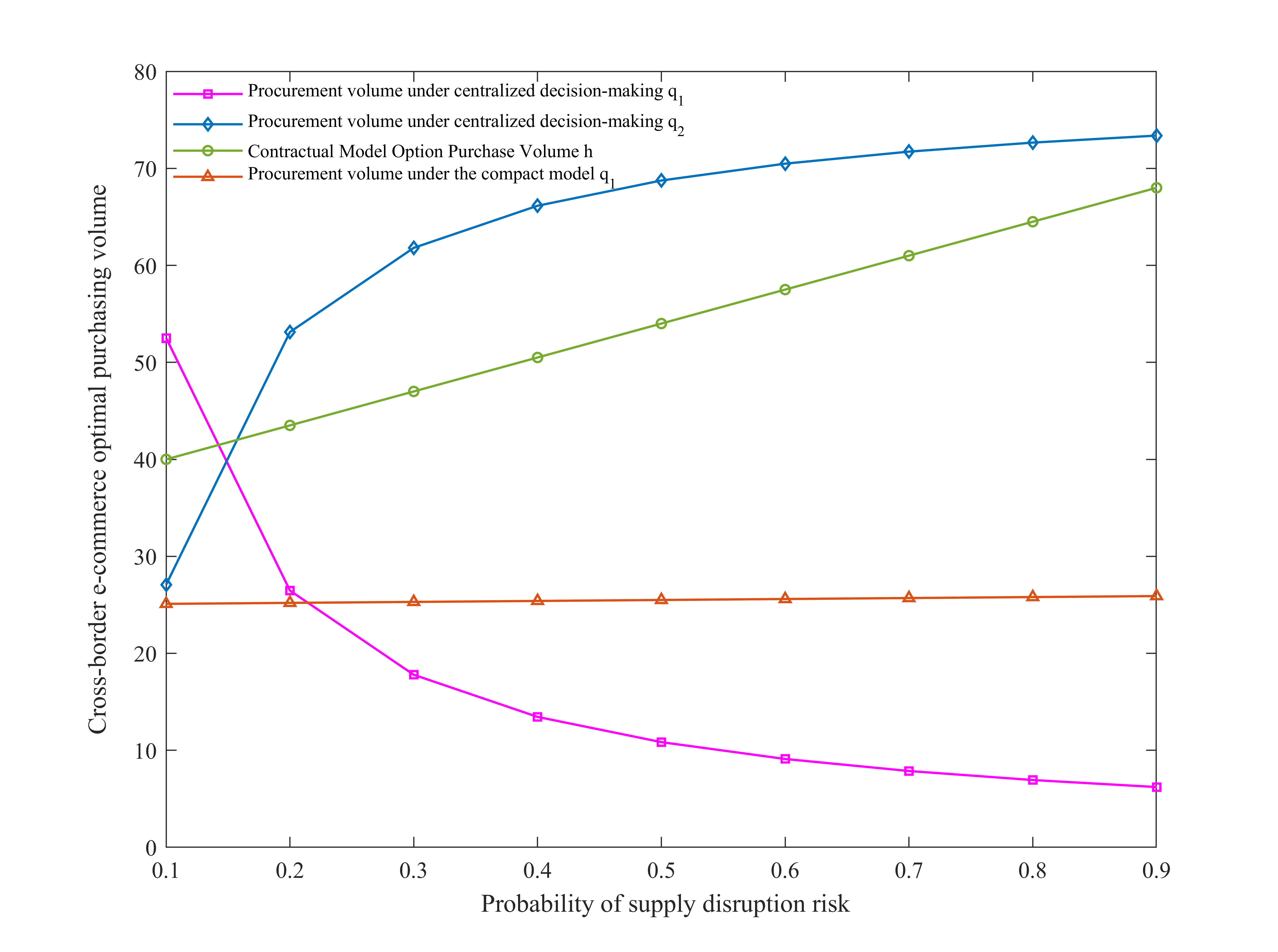

Supplement: S1 Appendix — (ZIP) [file pone.0309763.s001.zip › Fig2.tif]

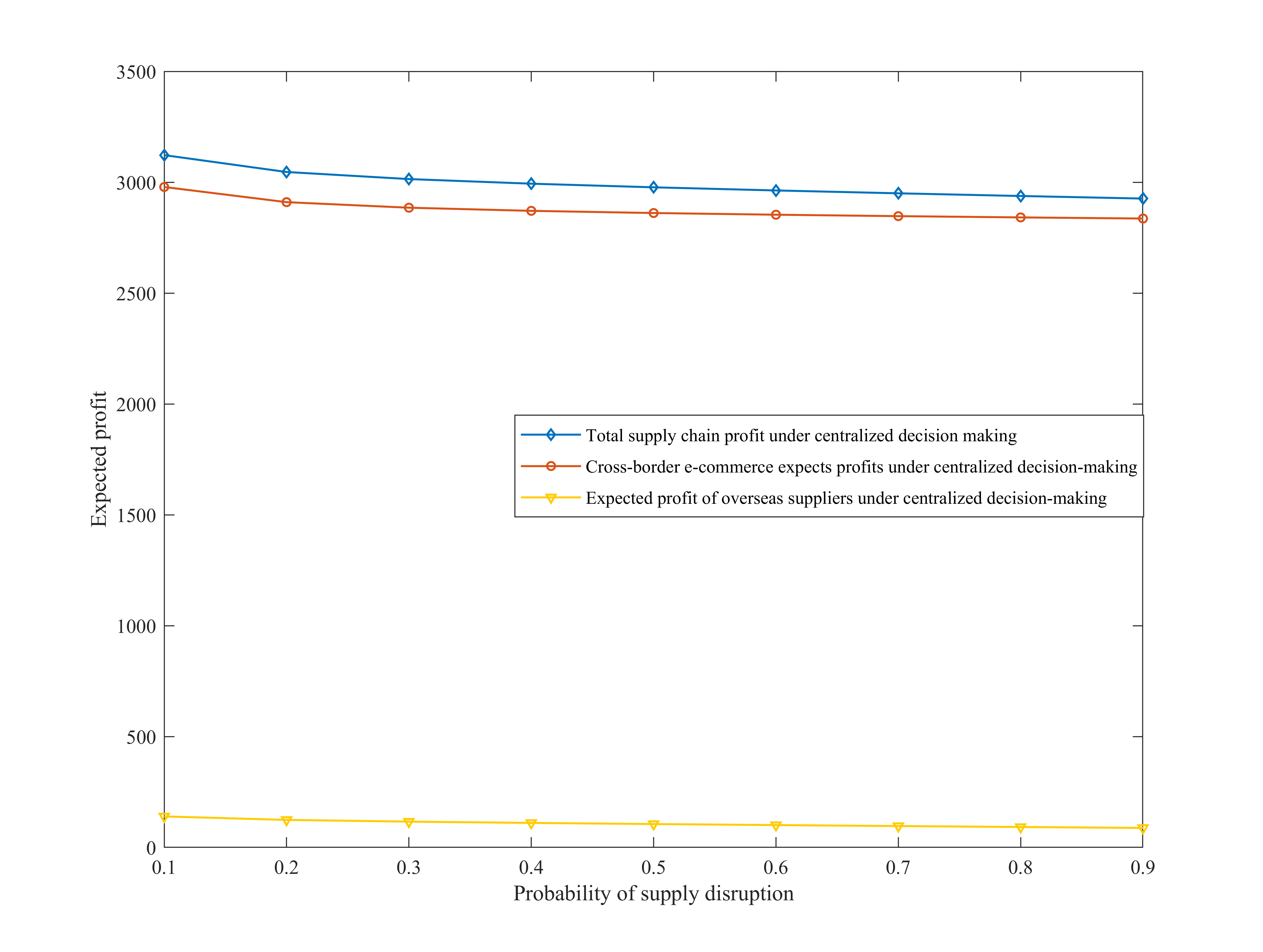

Supplement: S1 Appendix — (ZIP) [file pone.0309763.s001.zip › Fig3.tif]

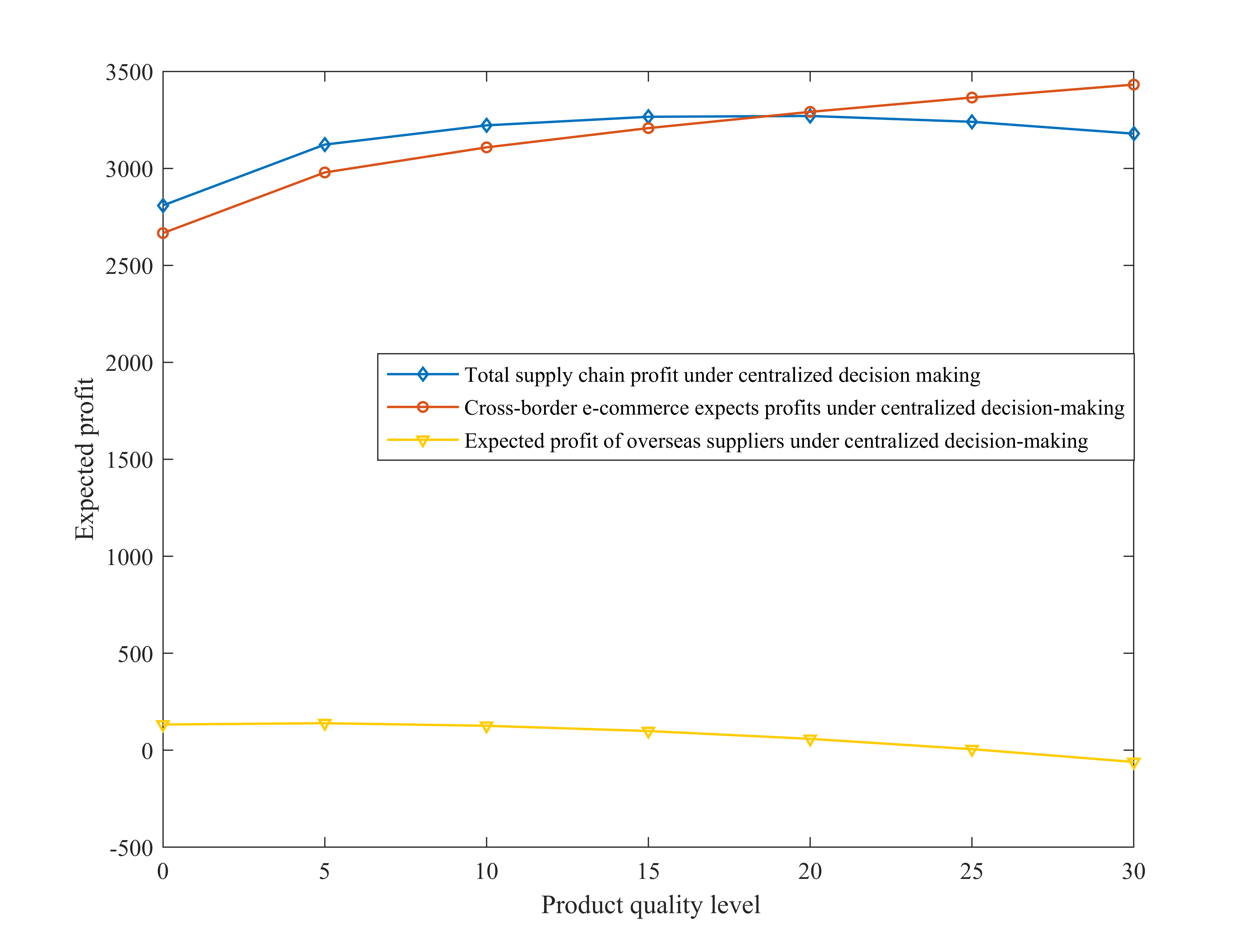

Supplement: S1 Appendix — (ZIP) [file pone.0309763.s001.zip › Fig4.tif]

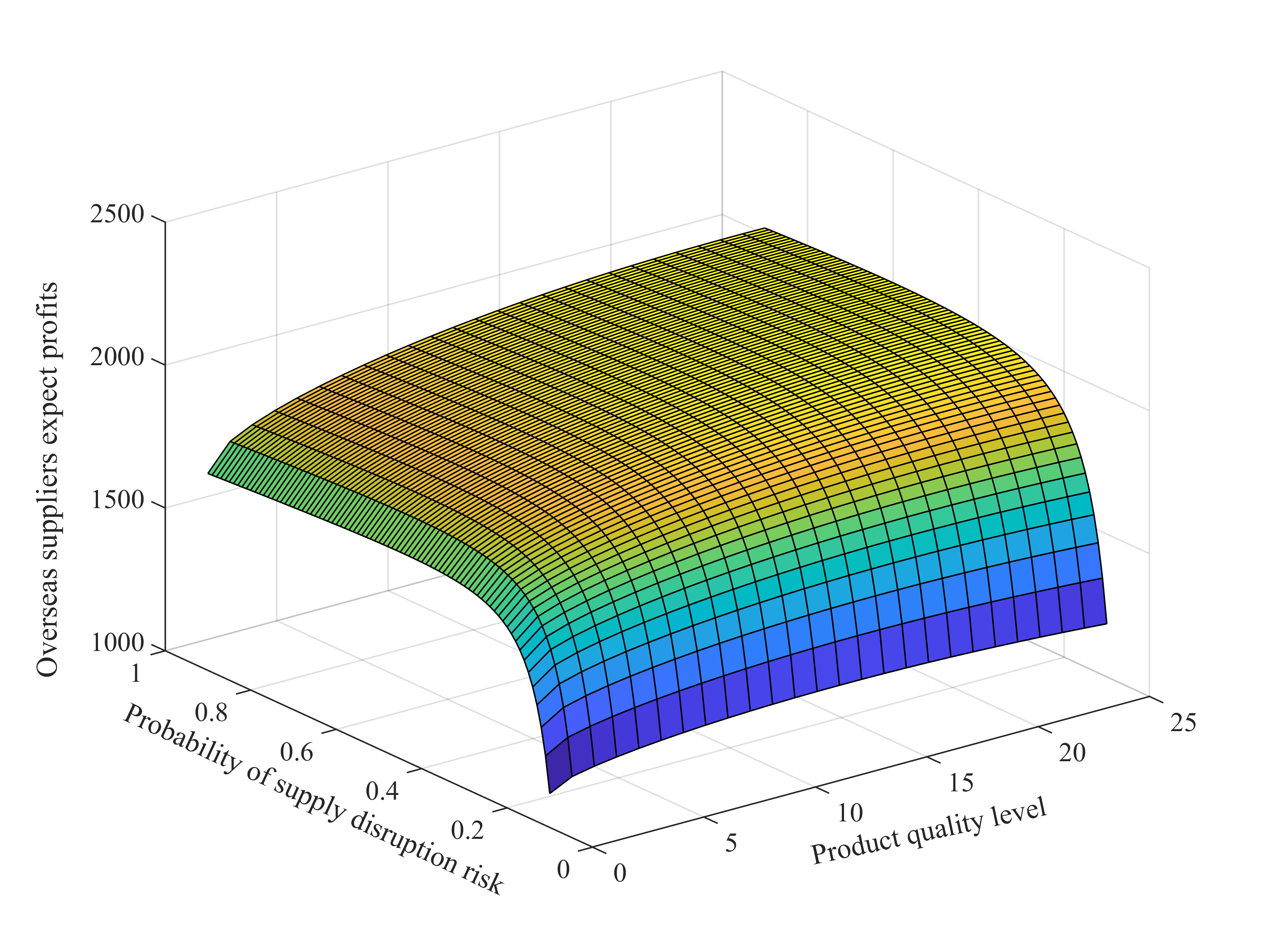

Supplement: S1 Appendix — (ZIP) [file pone.0309763.s001.zip › Fig5_a.tif]

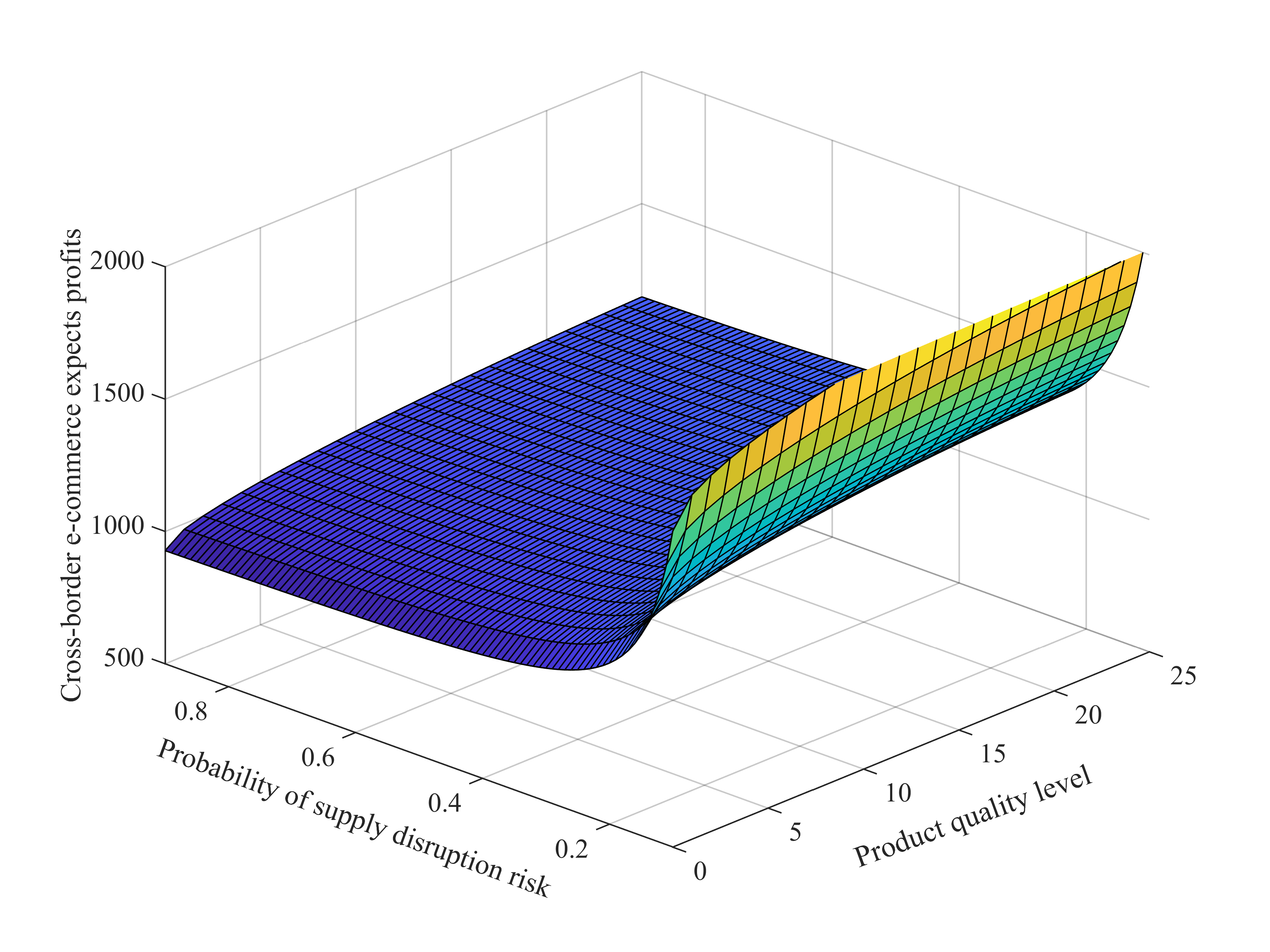

Supplement: S1 Appendix — (ZIP) [file pone.0309763.s001.zip › Fig5_b.tif]

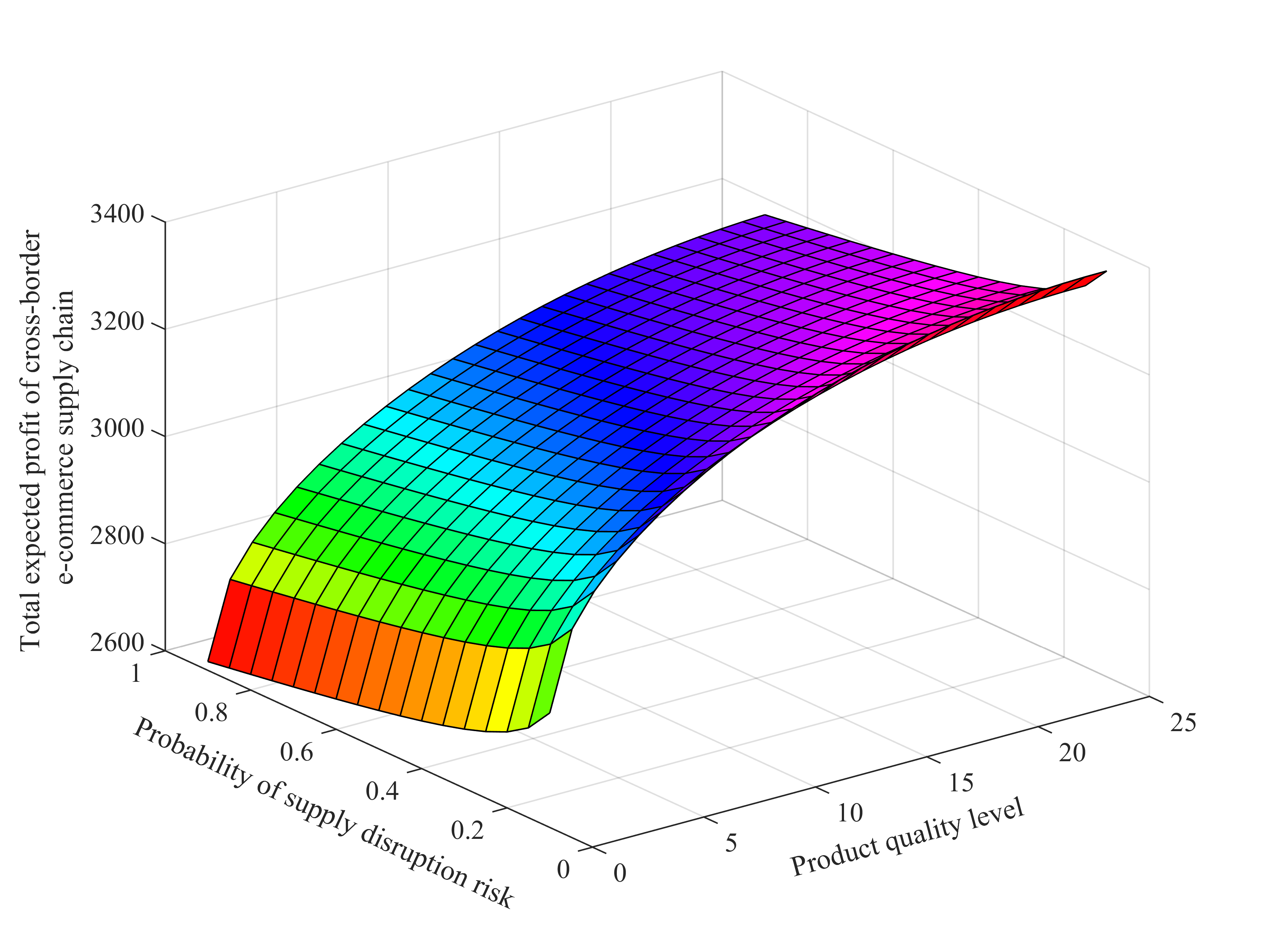

Supplement: S1 Appendix — (ZIP) [file pone.0309763.s001.zip › Fig5_c.tif]

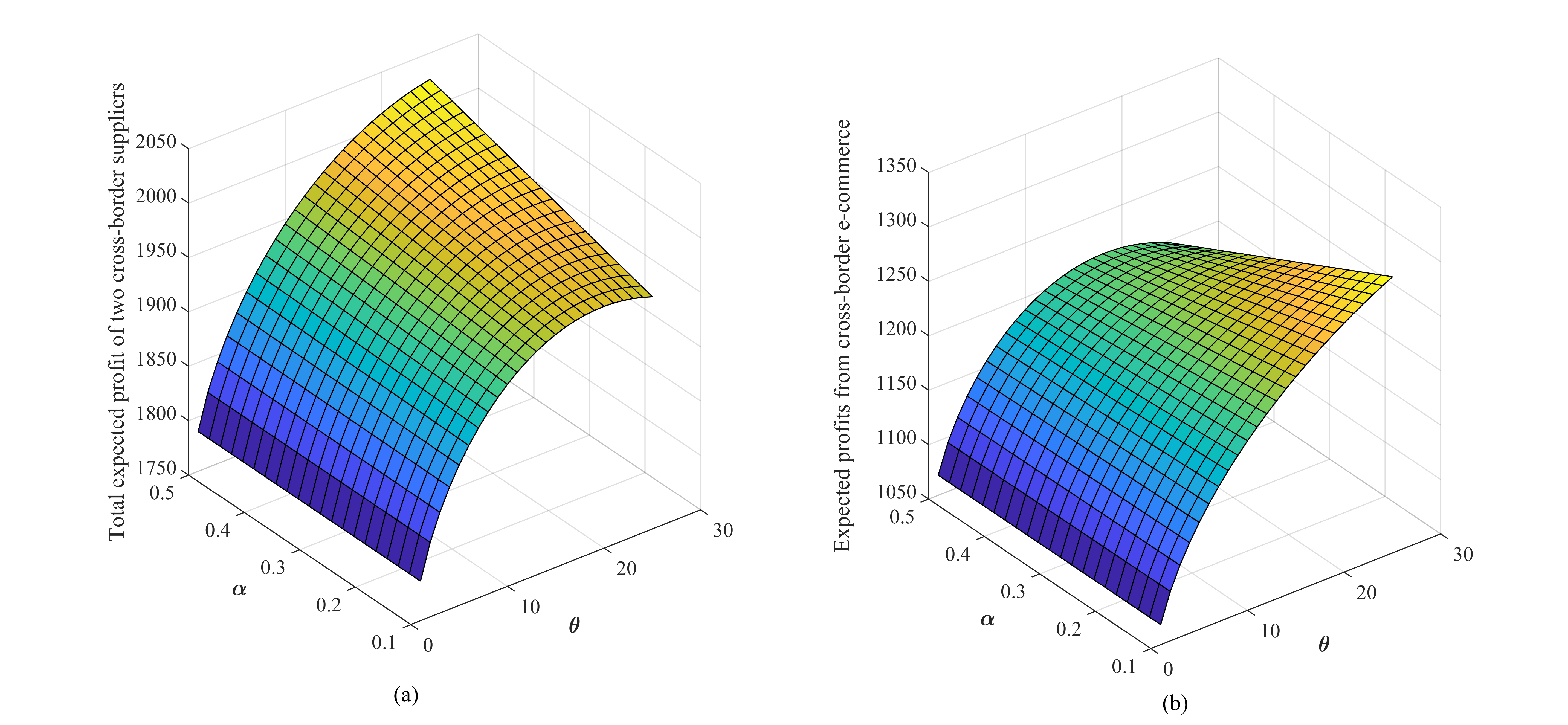

Supplement: S1 Appendix — (ZIP) [file pone.0309763.s001.zip › Fig6_a_b.tif]

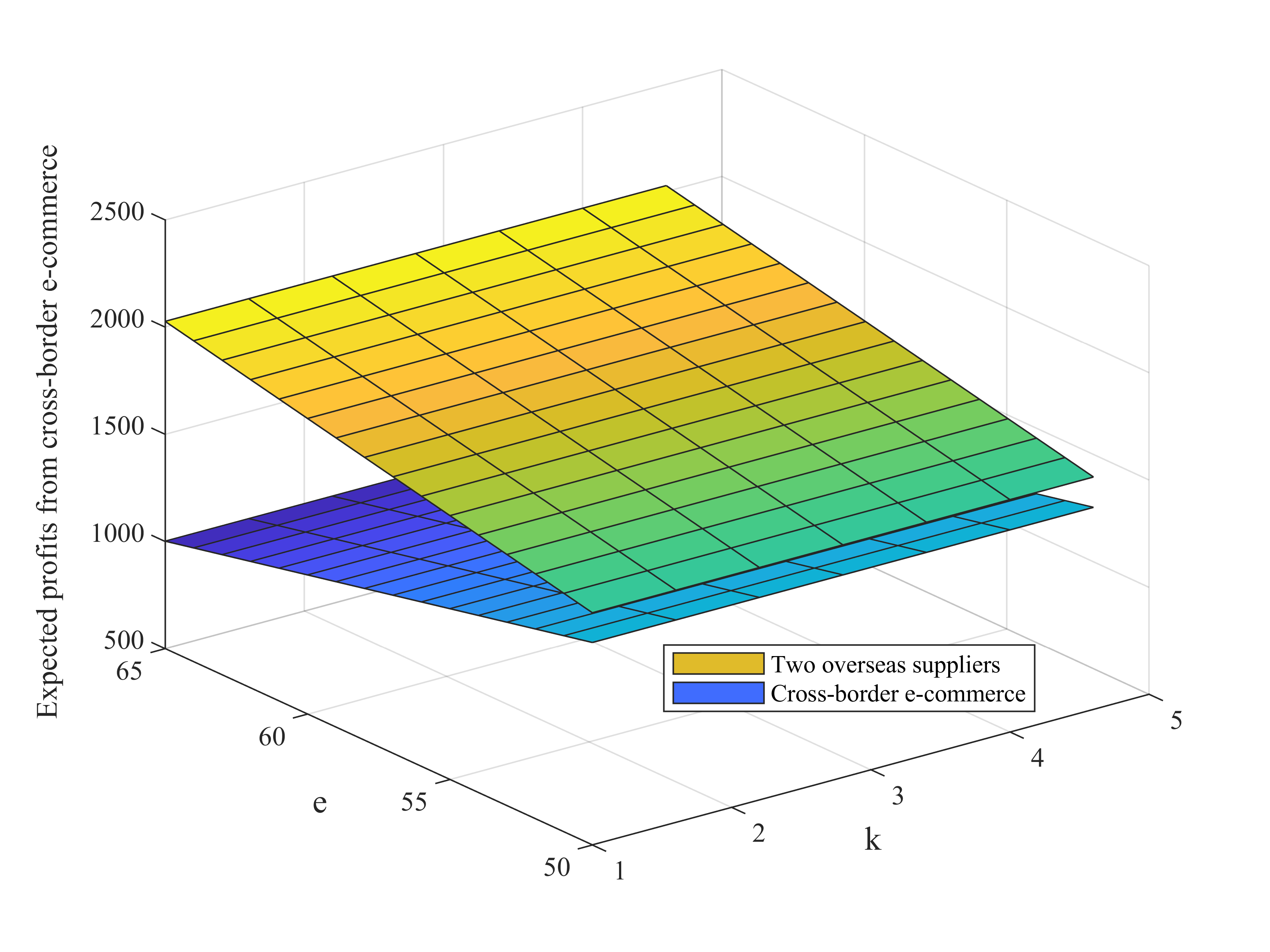

Supplement: S1 Appendix — (ZIP) [file pone.0309763.s001.zip › Fig7.tif]
