# Supplementary material for: Cross-border supply chain coordination of low-carbon agricultural products under the risk of supply uncertainty
Source: PLoS One. 2024 Oct 22;19(10):e0309763. doi: 10.1371/journal.pone.0309763 (PMC11495562; doi:10.1371/journal.pone.0309763)
Supplement: S1 Data — (DOCX) [file pone.0309763.s002.docx]

S1_Data

When the supply chain of cross-border e-commerce considers the risk of supply interruption and product quality control, there is a game among the participants. The optimal product quality level of overseas suppliers and the optimal purchase quantity of cross-border e-commerce depend on the selection of relevant parameters of the model. The above models are analyzed with examples, and the optimal strategies under different decision-making modes are compared. The influences of supply interruption risk probability, risk avoidance coefficient, product quality level and other parameters on the profit and utility of cross-border supply chain are analyzed, so as to verify whether options-quality cost-sharing contract can achieve supply chain coordination. In order to facilitate calculation, it is assumed that the market demand function is $F(X)=\eta\sqrt{\alpha}+\varepsilon$, the market random demand variable $\varepsilon$ follows the uniform distribution on the interval [1,100], and the product quality input costs of overseas suppliers are all $g(\alpha)=\frac{\mu\alpha^{2}}{2}=25\alpha^{2}$. According to the objective realistic environment, other relevant parameters of the model are set as follows: $p=90$, $w_{1}=60$, $w_{2}=65$, $c_{1}=20$, $c_{2}=25$, $v_{s}=2$, $v_{r}=4$, $\lambda=2$, $\eta=2$.
